# Responses of two marine top predators to an offshore wind farm

**DOI:** 10.1002/ece3.3389

**Published:** 2017-09-18

**Authors:** Gillian C. Vallejo, Kate Grellier, Emily J. Nelson, Ross M. McGregor, Sarah J. Canning, Fiona M. Caryl, Nancy McLean

**Affiliations:** ^1^ Natural Power Stirling UK; ^2^ Queen's Crescent Falkirk UK

**Keywords:** climate change, common guillemot, displacement, environmental impact assessment, harbor porpoise, marine mammal, renewables, seabird, spatial modeling, UK

## Abstract

Quantifying the likely effects of offshore wind farms on wildlife is fundamental before permission for development can be granted by any Determining Authority. The effects on marine top predators from displacement from important habitat are key concerns during offshore wind farm construction and operation. In this respect, we present evidence for no significant displacement from a UK offshore wind farm for two broadly distributed species of conservation concern: common guillemot (*Uria aalge*) and harbor porpoise (*Phocoena phocoena*). Data were collected during boat‐based line transect surveys across a 360 km^2^ study area that included the Robin Rigg offshore wind farm. Surveys were conducted over 10 years across the preconstruction, construction, and operational phases of the development. Changes in guillemot and harbor porpoise abundance and distribution in response to offshore wind farm construction and operation were estimated using generalized mixed models to test for evidence of displacement. Both common guillemot and harbor porpoise were present across the Robin Rigg study area throughout all three development phases. There was a significant reduction in relative harbor porpoise abundance both within and surrounding the Robin Rigg offshore wind farm during construction, but no significant difference was detected between the preconstruction and operational phases. Relative common guillemot abundance remained similar within the Robin Rigg offshore wind farm across all development phases. Offshore wind farms have the potential to negatively affect wildlife, but further evidence regarding the magnitude of effect is needed. The empirical data presented here for two marine top predators provide a valuable addition to the evidence base, allowing future decision making to be improved by reducing the uncertainty of displacement effects and increasing the accuracy of impact assessments.

## 1 INTRODUCTION

Offshore wind energy forms a significant part of international efforts to reduce atmospheric carbon dioxide emissions. To date, much of the development of offshore wind energy generation has occurred in Europe where there is a binding agreement for at least a 20% share of energy consumed to come from renewable sources by 2020 (Directive 2009/28/EC; European Commission 2008). Significant growth in the offshore wind industry is also expected in other key markets outside of Europe (Kaldellis & Kapsali, 2013). This increase in projected growth has led to concerns about the potential for offshore wind farms to negatively impact wildlife, including fish, marine mammals, and birds (e.g., Drewitt & Langston, 2006; Gilles, Scheidat, & Siebert, 2009; Wahlberg & Westerberg, 2005). Within Europe, the assessment of potential effects on wildlife is required as part of the application process for permission to construct and operate offshore wind farms. As a result, some projects have recently been canceled due to the population level implications arising from predicted negative effects upon ecological receptors (e.g., DECC 2012; Smith, 2014).

However, there is a significant element of uncertainty around the magnitude and consequences of effects from offshore wind farms on marine wildlife. This is the result, in part, of a lack of empirical data (Bailey, Brookes, & Thompson, 2014; Furness, Wade, & Masden, 2013). Such uncertainty can result in increased precaution in impact assessment (Masden, McCluskie, Owen, & Langston, 2015), leading to overly precautious conclusions and thus consenting decisions. As the industry expands globally, improving the evidence base and reducing the uncertainty inherent in these assessments will enable more informed decisions to be made (Hill & Arnold, 2012; Masden et al., 2015).

Predicted impacts on marine top predators may arise through effects such as anthropogenic noise pollution, direct collision with turbines, and wind farm avoidance (Boehlert & Gill, 2010; Gill, 2005; Inger et al., 2009). These effects will vary among species depending upon their specific sensitivities (Bailey et al., 2014; Dierschke, Furness, & Garthe, 2016; Furness et al., 2013). Species may avoid offshore wind farms in response to specific stimuli as a result of pile driving of turbine foundations, increased vessel activity, and/or the presence of turbines (Dolman & Simmonds, 2010; Fox & Petersen, 2006). Displacement can lead to habitat loss if individuals avoid offshore wind farms completely or habitat degradation if they do so only partially, even though habitats remain available (Bailey et al., 2010; Furness et al., 2013). Thus, displacement from offshore wind farms during construction and operation may result in the loss of key habitats for marine top predators, which in turn may impact individual survival and future productivity (Masden et al. 2010; Dähne et al., 2013).

Monitoring at constructed offshore wind farms in European waters to date has shown that displacement is likely to be site‐specific (Dähne et al., 2014; Dierschke et al., 2016; Leopold, Dijkman, & Teal, 2011; Petersen, Christensen, Kahlert, Desholm, & Fox, 2006; Scheidat et al., 2011; Teilmann & Carstensen, 2012). Over time, species that were initially displaced by the presence of an offshore wind farm may return (Petersen & Fox, 2007; Teilmann & Carstensen, 2012; Thompson et al., 2010). However, few empirical datasets exist to determine the extent of such responses (Bailey et al., 2014), despite the undisputable importance of an improved knowledge base regarding long‐term avoidance responses for marine spatial planning (Crowder & Norse, 2008).

Here, we analyzed abundance and distribution data collected during more than 10 years of monitoring at Robin Rigg offshore wind farm (OWF) in the UK to test for evidence of displacement (defined as a reduced number of individuals occurring within, or immediately adjacent to, an offshore wind farm) of two marine top predators: common guillemot (*Uria aalge*; hereafter guillemot) and harbor porpoise (*Phocoena phocoena*). Both guillemot and harbor porpoise are broadly distributed in European waters and are frequently identified as important species in offshore wind farm impact assessments due to their protected status under national and international legislation (Bailey et al., 2014; Furness et al., 2013).

## 2 MATERIALS AND METHODS

### 2.1 Study site

The Robin Rigg OWF is situated within the Solway Firth in the northern Irish Sea. The Solway Firth is a shallow estuary characterized by high wave exposure and moderate tidal currents, resulting in a highly dynamic environment (Gray & Elliott, 2009; Walls, Canning, et al., 2013). As such, the Solway Firth has long been recognized as being of high environmental importance with several key protected areas (JNCC 2015; SNH 2014).

**Figure 1:**
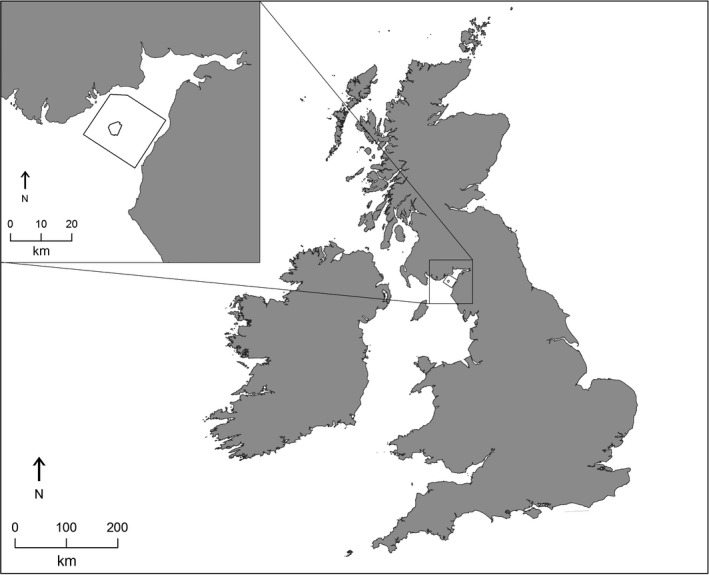
Location of the Robin Rigg offshore wind farm in the context of the UK and the Solway Firth (inset). Small polygon shows the offshore wind farm footprint. Larger polygon shows the study area

The Robin Rigg OWF comprises 60 turbines, covers 13 km^2^, and is situated immediately to the north of the boundary between Scottish and English territorial waters (Figure 1). Consent for the scheme was granted by the Scottish Executive in March 2003. Construction began in December 2007, with piling and cable laying activities completed by February 2010 (Walls, Canning, et al., 2013). Full commercial operation began in April 2010, and the scheme is the first commercial offshore wind farm in Scottish territorial waters.

In accordance with the consent from Scottish Ministers, a Marine Environment Monitoring Programme (MEMP) was developed in conjunction with the Robin Rigg Management Group (RRMG) prior to construction (MEMP 2004).

### 2.2 Data collection

Boat‐based line transect surveys were used to collect guillemot and harbor porpoise abundance and distribution data across a 360 km^2^ study area including the Robin Rigg OWF during three development phases: preconstruction, construction, and operation (Table 1). Full details of the survey schedules can be found in the Supporting Information (Appendix S1).

**Table 1:** Survey effort (numbers in brackets show survey effort within the wind farm footprint), number of observations, and observations per km survey effort (within respective strip widths) for guillemot and harbor porpoise across the Robin Rigg offshore wind farm study area during three development phases: preconstruction, construction, and operation (for full details of survey schedules see Appendix S1)

	Guillemot			Harbor porpoise		
Development phase	Total survey effort (km)	Number of observations	Observations per km (±standard error)	Total survey effort (km)	Number of observations	Observations per km (±standard error)
Preconstruction	4,283 (181)	3,803	1.48 (±0.056)	2,592 (106)	139	0.054 (±0.006)
Construction	2,833 (105)	2,027	1.19 (±0.054)	2,636 (100)	70	0.027 (±0.004)
Operation	3,746 (153)	2,974	1.32 (±0.049)	3,783 (158)	228	0.060 (±0.005)
Total	10,862 (439)	8,804	1.35 (±0.031)	9,011 (364)	437	0.048 (±0.003)

A number of vessels were used throughout the bird and marine mammal surveys specified within the MEMP, with viewing platforms ranging from 3.5 to 4.5 m above sea level. Although below the minimum platform height of 5 m recommended by Camphuysen, Fox, Leopold, and Petersen (2004) and Maclean, Wright, Showler, and Rehfisch (2009), these vessels provided suitable viewing platforms without restricting access to the entire study area; vessels with higher viewing platforms had deeper drafts and so were unable to navigate across the shallow sandbanks that run through the Solway Firth. The level of survey effort undertaken at each viewing platform height is provided in the Supporting Information (Appendix S2). During each survey, the vessel travelled along ten parallel transects, each approximately 18 km long and spaced 2 km apart. This separation distance was chosen to gather a representative sample of data for each species, while minimizing the risk of double‐counting resulting from the survey vessel displacing individuals from one transect into another.

Beaufort sea state was recorded at the start of each transect and then every 15 min thereafter. Vessel location was recorded every 30 s on a hand‐held global positioning system (GPS) unit. The time piece used for recording sightings was synchronized to the hand‐held GPS used for recording transect tracks, allowing the approximate position of each observation to be determined. Detection was undertaken by eye, and high‐quality binoculars were used to confirm species identity. Distance of each sighting from the vessel was determined using a rangefinder measuring stick (Heineman, 1981).

#### 2.2.1 Guillemot

Best practice European Seabirds at Sea (ESAS) methods were followed (Camphuysen et al., 2004; Maclean et al., 2009; Tasker, Jones, Dixon, & Blake, 1984), using a transect strip width of 600 m, with two surveyors observing out to 300 m on either side of the vessel. For each guillemot, observation, time, number of individuals and behavior (‘in flight’ or ‘on sea’) were recorded as a minimum. Time was recorded to the nearest minute.

#### 2.2.2 Harbor porpoise

The area of sea spanning 180° to the front and either side of the vessel (90° either side of the transect line) was scanned by a single surveyor and a strip width of 1 km was used. For each harbor porpoise sighting, time and number of individuals were recorded as a minimum. Data were collected in sea states zero to five.

### 2.3 Data analysis

To quantitatively compare underlying changes in relative guillemot and harbor porpoise abundance and distribution across preconstruction, construction, and operational phases of the Robin Rigg OWF, the spatial distribution of individual observations per km was modeled. Transect lines for all surveys were divided into 600 m × 600 m segments for guillemot and 1 km × 1 km segments for harbor porpoise. Guillemot and harbor porpoise observations were then assigned to these segments. Only guillemots recorded ‘on sea’ were included in the analysis; birds ‘in flight’ were excluded since it was not known whether these individuals were actively using the habitat within the study area or simply passing through (Camphuysen et al., 2004). Multiple observations within a single segment were summed to give the total number of individuals observed per segment. Latitude and longitude were extracted for the midpoint of each segment using ArcGIS (version 10.2) and were converted to the Universal Transverse Mercator (UTM) coordinate system (zone 30N) for analysis. For interpretation of model outputs, the “study area” is defined as the area covered by the survey transects and the wind farm “footprint” refers to a smaller polygon within the study area which bounds the turbines (Figure 1).

Distance sampling is often used to convert the rate of observations along line transects into estimated abundances by constructing a detection function which can account for decreasing detectability with increasing distance from an observer (Buckland et al., 2001). Due to the methods of data collection, distance sampling could not be undertaken. However, as our aims were to investigate changes in the relative abundance of birds and marine mammals rather than absolute numbers, modeling uncorrected observations is as informative.

#### 2.3.1 Guillemot

The guillemot model consisted of a negative binomial generalized additive mixed model (NB GAMM) with number of guillemot per segment as the response variable. Explanatory variables included development phase and a two‐dimensional smooth of longitude and latitude which was allowed to vary by development phase (i.e., distinct smooths were generated for each development phase). The two‐dimensional smooth of longitude and latitude acts as a proxy for any underlying spatio‐temporal covariates that are driving guillemot distribution. Surveys, and transect within survey, were incorporated as random effects to account for spatio‐temporal dependency inherent within the data. The model was used to predict relative abundance across the study area on a 0.36 km^2^ scale.

#### 2.3.2 Harbor porpoise

The harbor porpoise dataset contained a large percentage of zeros and data were overdispersed when modeled using Poisson or negative binomial distributions. The model for harbor porpoise therefore consisted of a zero‐inflated Poisson generalized additive mixed effects model (ZIP GAMM) with number of harbor porpoise per segment as the response variable. Explanatory variables for the Poisson (count) part of the model included development phase and a two‐dimensional smooth of longitude and latitude which was allowed to vary by development phase (i.e., distinct smooths were generated for each development phase). As for guillemot, the two‐dimensional smooth of longitude and latitude acts as a proxy for any underlying spatio‐temporal covariates that may be driving the distribution of the animals. The binary part of the model was used to account for additional zeros resulting from “sampling error” (i.e., segments where individuals were not present during the survey despite the segment providing suitable conditions for occupation), “unavailability” (i.e., if individuals were present within a segment but were underwater and therefore not observed), and “detectability” (i.e., if an individual was present but not observed). The latter was more likely in higher sea states. As such, survey conditions were included as an explanatory variable in the binary part of the harbor porpoise model. Survey conditions were modeled as either ‘good’ (sea states of 0–2) or ‘poor’ (sea states of 3–5) in order to simplify the model and balance the covariate (survey effort was lower at sea states 0, 1 and 5; Appendix S3, Supporting Information). As with the guillemot model, survey and transect within survey were incorporated into the Poisson part of the model as random effects. The model was used to predict relative abundance across the site on a 1 km^2^ scale.

Both models were undertaken using Markov chain Monte Carlo (MCMC) Bayesian inference using JAGS (Plummer, 2003) implemented through the R package ‘R2jags’ (Yu‐Sung & Masanao, 2015) in R version 3.2.2 (R Core Team 2015) and were implemented based on the analysis carried out in Zuur, Canning, Lye, and Walls (2014). Full details of model formulation and parameterization can be found in the Supporting Information (Appendix S4).

## 3 RESULTS

### 3.1 Guillemot

**Figure 2:**
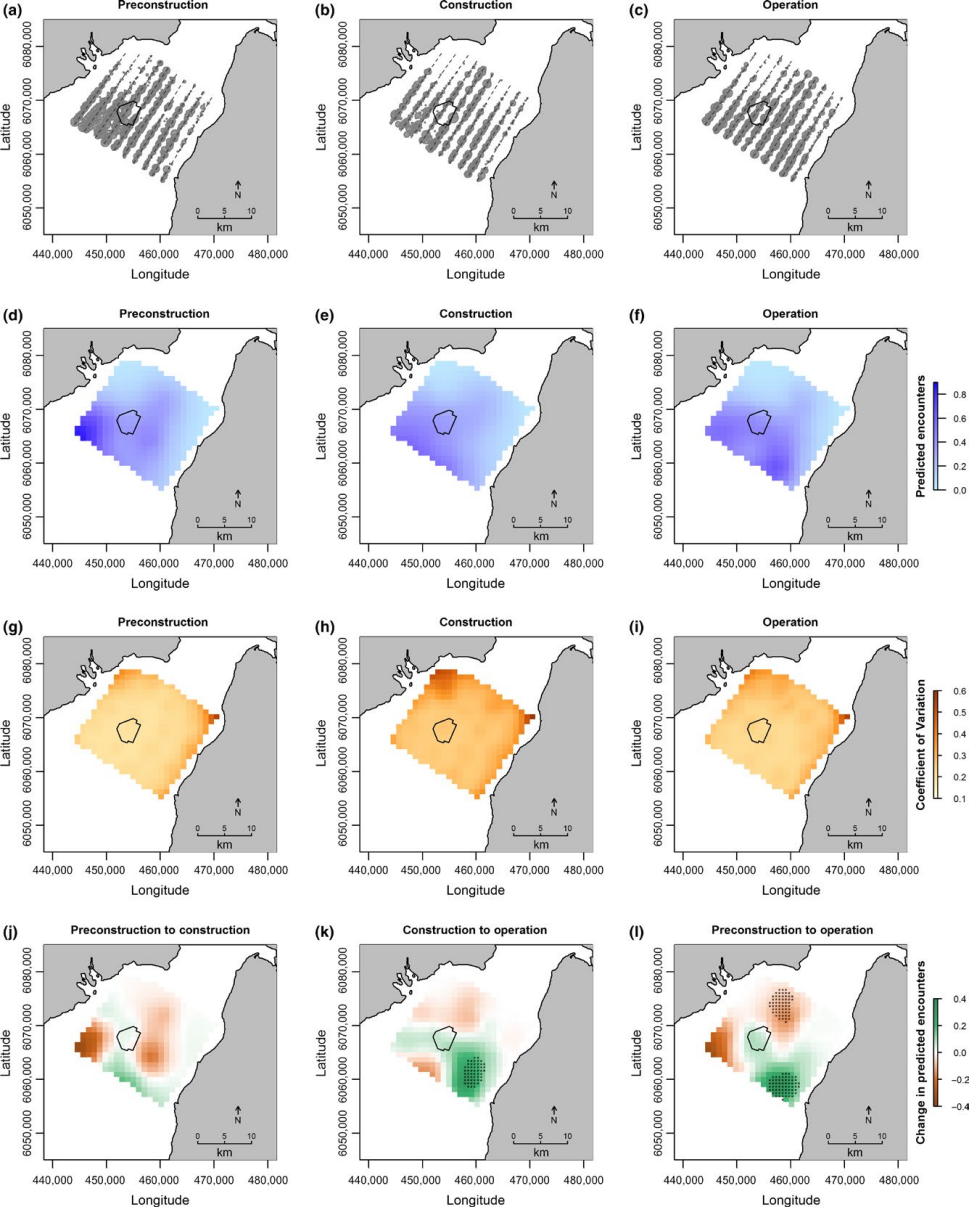
Top row: guillemot observations during (a) preconstruction, (b) construction, and (c) operation. Circle size indicates number of individuals recorded per observation (range: 1–39), and dashed lines represent indicative transect routes. Upper middle row: predicted number of guillemot per segment during (d) preconstruction, (e) construction, and (f) operation. Lower middle row: confidence in predicted number of guillemot per segment (standard deviation expressed as a proportion of the mean) during (g) preconstruction, (h) construction, and (i) operation. Bottom row: change in predicted number of guillemot per segment between (j) preconstruction and construction, (k) construction and operation, and (l) preconstruction and operation. Asterisks represent significant changes, that is, cells in which 95% credible intervals of the predicted values do not overlap. Data are shown using the Universal Transverse Mercator (UTM) coordinate system (zone 30N)

Guillemots were observed across the study area during all three development phases, both within and outside the Robin Rigg OWF (Figure 2). However, the number of guillemot observations across the entire study area was highest during the preconstruction phase and lowest during the construction phase (Table 1).

Development phase did not predict relative guillemot abundance across the study area as a whole (Table 2), and no change was detected within the footprint of the Robin Rigg OWF across the three development phases (Figure 2j, 2k and 2l). Model confidence across the footprint of the Robin Rigg OWF was high (Figures 2g, 2h and 2i). No differences were detected in the spatial distribution of guillemots across the study area between the preconstruction and construction phases (Figure 2j). There was a decline in relative guillemot abundance to the north of the site from the preconstruction phase to the operational phase (Figure 2l). Relative guillemot abundance increased in the south of the site from the construction to operational phases (Figures 2d, 2e, and 2k), and abundance in this area was higher during the operational phase than during the preconstruction phase (Figures 2d, 2f and 2l).

**Table 2:** Parameter estimates for nonsmooth components of the guillemot model and their significance

	Raw parameters			Response level parameters			
		Credible intervals			Credible intervals		
Model parameter	Estimate	2.5%	97.5%	Estimate	2.5%	97.5%	Significant*
Preconstruction versus construction	−0.371	−1.249	0.495	0.690	0.287	1.640	No
Construction versus operation	0.346	−0.503	1.198	1.414	0.605	3.314	No
Preconstruction versus operation	−0.025	−0.861	0.830	0.975	0.423	2.294	No

*Significant predictors are defined as those for which the 95% credible intervals of the raw parameters do not bound zero.

### 3.2 Harbor porpoise

**Figure 3:**
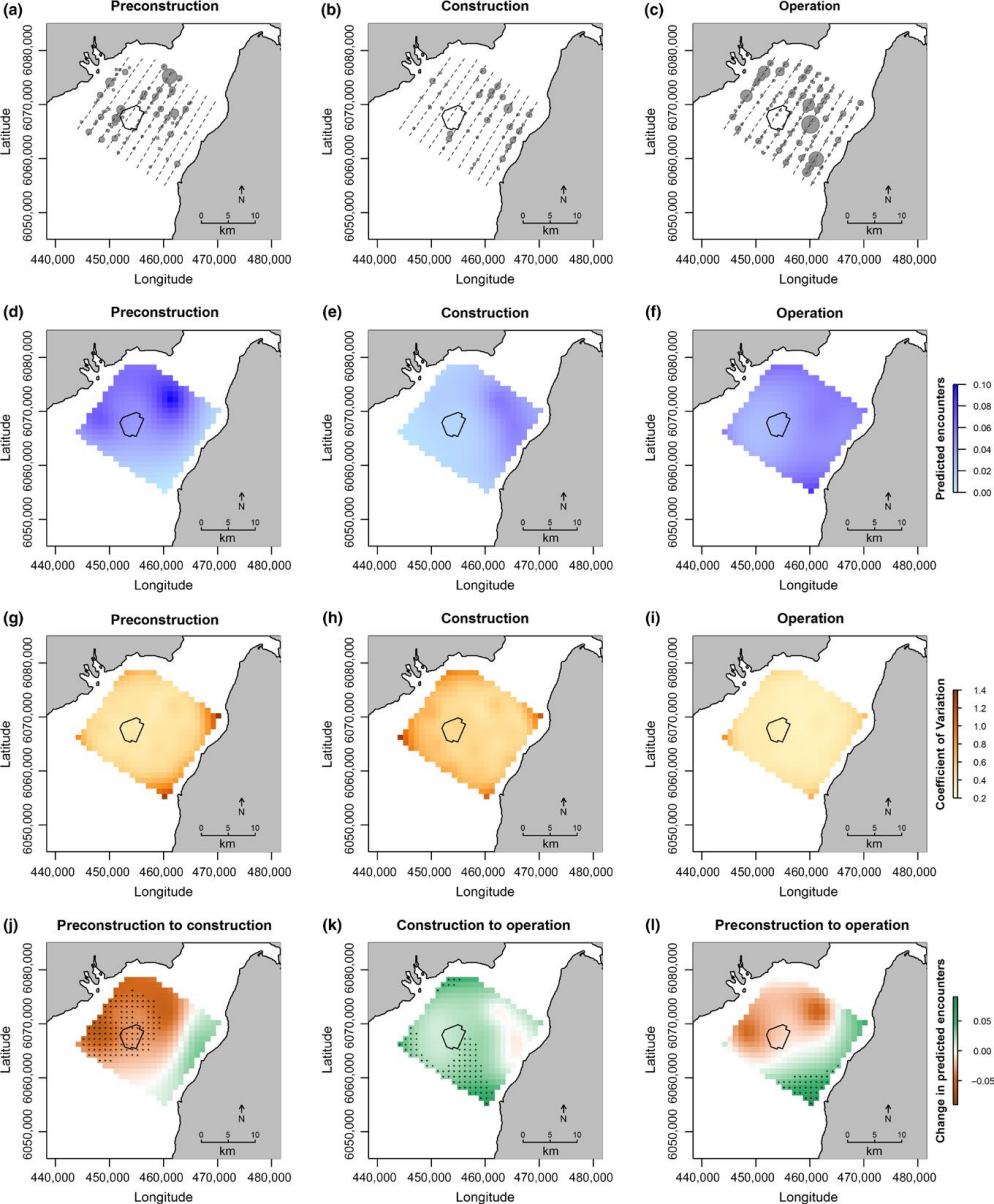
Top row: harbor porpoise observations during (a) preconstruction, (b) construction and (c) operation. Circle size indicates number of individuals recorded per observation (range: 1–6), and dashed lines represent indicative transect routes. Upper middle row: predicted number of harbor porpoise per segment during (d) preconstruction, (e) construction, and (f) operation. Lower middle row: confidence in predicted number of harbor porpoise per segment (standard deviation expressed as a proportion of the mean) during (g) preconstruction, (h) construction, and (i) operation. Bottom row: change in predicted number of harbor porpoise per segment between (j) preconstruction and construction, (k) construction and operation, and (l) preconstruction and operation. Asterisks represent significant changes, that is, cells in which 95% credible intervals of the predicted values do not overlap. Data are shown using the Universal Transverse Mercator (UTM) coordinate system (zone 30N)

Harbor porpoise were observed throughout the Robin Rigg study area during all three development phases. However, harbor porpoise were not recorded within the footprint of the Robin Rigg OWF during the construction phase (Figure 3). Harbor porpoise observations were also much less frequent across the rest of the study area during the construction phase, with approximately twice as many individuals observed per km survey effort during the preconstruction and operational phases (Table 1).

Development phase did not predict overall relative harbor porpoise abundance (Table 3). However, relative harbor porpoise abundance decreased within and to the northwest of the Robin Rigg OWF footprint from the preconstruction phase to the construction phase (Figure 3d, 3e, and 3j). There was no difference in relative abundance of harbor porpoise within the wind farm footprint between the preconstruction and operational phases (Figure 3l) and relative harbor porpoise abundance was higher in the south of the study area during the operational phase than the preconstruction and construction phases (Figures 3d, 3e, 3f, and 3l). The model also suggests an increase in relative harbor porpoise abundance in the northernmost and westernmost tips of the study area from the construction phase to the operational phase (Figures 3e, 3f and 3l). The probability of zero observations was significantly higher during ‘poor’ survey conditions than during ‘good’ survey conditions (Table 3).

**Table 3:** Parameter estimates for the nonsmooth components of the harbor porpoise model and their significance

	Raw parameters			Response level parameters			
		Credible intervals			Credible intervals		
Model parameter	Estimate	2.5%	97.5%	Estimate	2.5%	97.5%	Significant*
Preconstruction versus construction	−0.874	−2.299	0.479	0.417	0.100	1.615	No
Construction versus operational	0.873	−0.325	2.179	2.395	0.723	8.840	No
Preconstruction versus operational	<0.001	−2.300	0.479	0.999	0.343	2.935	No
Good sea state versus poor sea state**	1.190	0.820	1.551	0.767	0.694	0.825	Yes

*Significant predictors are defined as those for which the 95% credible intervals of the raw parameters do not bound zero. **Survey conditions were modeled as either ‘good’ (sea states of 0–2) or ‘poor’ (sea states of 3–5)

## 4 DISCUSSION

Improving the evidence base and reducing uncertainty surrounding the potential effects on wildlife from offshore wind energy developments will enable more informed decisions to be made as the industry expands globally (Hill & Arnold, 2012; Masden et al., 2015). However, estimating the impact of displacement by offshore wind farms on broadly distributed marine top predators is problematic due to the significant technical challenges and costs associated with monitoring and quantifying the responses of marine wildlife to offshore wind farms (Bailey et al., 2014; Masden et al., 2015). Here, we have provided evidence for no significant displacement of two protected marine top‐predator species from an offshore wind farm in UK waters.

While survey effort within the wind farm footprint was constrained by the relatively small size of the wind farm and best practice for separation distance among transects (Camphuysen et al., 2004; Table 1), any localized changes large enough to be biologically significant should have been detected. Indeed, a significant reduction in relative harbor porpoise abundance both within and surrounding the Robin Rigg OWF during the construction phase was detected. However, no significant difference was detected between the preconstruction and operational phases, indicating that this local displacement was short‐term and restricted to the construction phase. In contrast, relative guillemot abundance remained similar within the Robin Rigg OWF across development phases. However, significant changes in modeled guillemot distribution were detected elsewhere within the study area between preconstruction and operation. Both guillemot and harbor porpoise were present across the Robin Rigg study area throughout all three development phases, providing evidence for no large‐scale displacement during construction and operation.

Short‐term displacement from the Robin Rigg OWF during the construction phase by harbor porpoise is consistent with findings at the Danish Horns Rev OWF, the Dutch Egmond aan Zee OWF, and the German Alpha Ventus OWF where harbor porpoise abundance increased following the construction phase (Dähne et al., 2014; Scheidat et al., 2011; Teilmann, Tougaard, & Carstensen, 2006). This short‐term displacement most likely resulted from increased levels of anthropogenic noise due to pile driving during the construction phase (Brandt, Diederichs, Betke, & Nehls, 2011). Several studies have demonstrated harbor porpoise avoidance behavior in response to pile driving activity at distances of up to 25 km (Dähne et al., 2013; Tougaard, Carstensen, Teilmann, Skov, & Rasmussen, 2009). At Robin Rigg OWF, avoidance behavior occurred at distances of less than 18 km, with harbor porpoise still present across the wider study area during the construction phase. The quick return of harbor porpoise to the study area following the cessation of construction activities at Robin Rigg OWF may be linked to habitat quality, with individuals more likely to show prolonged avoidance from offshore wind farms situated in less favorable habitat with poor prey availability (Scheidat et al., 2011).

To date, prolonged avoidance of an offshore wind farm by harbor porpoise has only been documented at the Nysted OWF, Denmark, where abundance did not return to preconstruction levels (Teilmann & Carstensen, 2012). Displacement during construction was likely to be associated with pile driving activity, but the slow return of harbor porpoise to the area during operation may be linked to habitat quality if habitat in the vicinity of Nysted OWF was less important to harbor porpoise than that elsewhere (Scheidat et al., 2011; Teilmann & Carstensen, 2012). Furthermore, harbor porpoise density in the Baltic Sea is relatively low compared to other European waters (e.g., 0.28 individuals per km^−2^; Hammond et al., 2013), which may indicate that individuals are not constrained by competition to forage within Nysted OWF.

Although changes in relative guillemot abundance were detected in some areas of the Robin Rigg OWF study area among the three development phases, the number of guillemot per segment within the OWF footprint remained comparable throughout all three development phases, providing no evidence of guillemot displacement. It is important to note that given the apparent lack of displacement, guillemots may be confronted with the risk of colliding with wind turbines at Robin Rigg OWF (Brabant, Vanermen, Stienen, & Degraer, 2015). However, very few guillemots were recorded flying through the footprint of the OWF throughout operational monitoring, with the majority of these birds (*c*.98%) flying below the rotor‐swept area. Thus, potential collision risk is very low (Walls, Pendlebury, et al., 2013).

The extent of guillemot avoidance from constructed offshore wind farms is highly variable (Dierschke et al., 2016). In the southern North Sea, guillemot density was significantly reduced after construction within Thorntonbank OWF (68% reduction) and Bligh Bank OWF (75% reduction; Vanermen, Courtens, Van de walle, Verstraete, & Steinen, 2016). In contrast, no effect of wind farm construction on guillemot abundance was found at Thanet OWF, which is also located in the southern North Sea (Ecology Consulting, 2012; Percival, 2013). Postconstruction monitoring at North Hoyle OWF, in the north Irish Sea, showed a 55% increase in the number of guillemot within the operational wind farm compared to preconstruction (PMSS, 2007).

The reasons for such variable responses are likely to be site‐specific (Dierschke et al., 2016; Leopold et al., 2011; Petersen et al., 2006). At the neighboring Princess Amalia and Egmond aan Zee offshore wind farms off the coast of the Netherlands significant displacement was found following construction (Leopold, van Bemmelen, & Zuur, 2013). The extent of displacement may have been influenced by the configuration of these offshore wind farms, with guillemot appearing to show stronger displacement from the Princess Amalia OWF which has a higher turbine density than Egmond aan Zee (Leopold et al., 2013). However, turbine density at Robin Rigg OWF (4.6 turbines/km^2^) is comparable to that at the Princess Amalia OWF (4.3 turbines/km^2^), indicating that a higher turbine density alone is unlikely to induce stronger displacement behaviors. Guillemots are only present in the Princess Amalia OWF during the winter (Leopold et al., 2013), when this species is known to range widely throughout the North Sea (Stone et al., 1995). As such, guillemots here may be more flexible in terms of habitat choice, compensating for any habitat loss by moving elsewhere to forage (Dierschke et al., 2016). In contrast, guillemots at Robin Rigg OWF may be constrained to foraging within the Solway Firth during the summer due to the proximity of breeding colonies (Orians & Pearson, 1979). Indeed, guillemot abundance was higher across the study area during the breeding season (Appendix S5, Supporting Information), but neither month nor season could be included in the model since these covariates were collinear with phase. A limitation of the current study is that the breeding season was underrepresented in the construction phase compared to the preconstruction and operational phases due to the relatively short period over which construction of the Robin Rigg OWF occurred. As a result, guillemot avoidance behavior during the breeding season during the construction phase could have occurred undetected. However, the comparison of relative guillemot abundance between the preconstruction and operational phases is robust meaning that any longer‐term avoidance behavior would have been detected.

Attributing changes in abundance and distribution directly to offshore wind farms is complicated by high levels of spatio‐temporal variation in the presence of highly mobile and widely distributed top predators in the marine environment (Maclean, Rehfisch, Skov, & Thaxter, 2013). For example, Teilmann et al. (2006) and Dähne et al. (2014) attributed changes in harbor porpoise abundance during offshore wind farm operation to wide‐scale variation in harbor porpoise abundance across the region (e.g., Hammond et al., 2013), rather than being an effect of offshore wind farm construction and operation. Significant localized changes in both guillemot and harbor porpoise distribution were modeled across the Robin Rigg study area during the three development phases which are likely to be independent of the Robin Rigg OWF. The Robin Rigg OWF is situated in the Solway Firth which is a highly dynamic environment characterized by mobile sediments brought into the area from the Irish Sea. Indeed, the original sandbanks present within the Solway Firth during baseline surveys undertaken in 2001 are known to have shifted in a southerly direction during the course of construction and operational monitoring (Malcolm, Lancaster, & Walker, 2013; ABP, 2015). Fish abundance data collected within the Solway Firth provide evidence that the distribution of nonmigratory fish moved in the same direction during the course of the bird and marine mammal surveys, presumably as these species followed shifting water channels through the estuary (Malcolm et al., 2013). It is therefore possible that changes in relative guillemot and harbor porpoise abundance and distribution across the three development phases reflect variation in prey distribution, given that both guillemot and harbor porpoise abundance increased significantly in the south of the study area between preconstruction and operation. Prey availability might thus be important in explaining contradictory study outcomes from operational monitoring and consideration of this may be required when predicting potential displacement effects from future offshore wind farm developments.

Overall, our results suggest that there has been no lasting displacement of guillemot or harbor porpoise attributable to the construction and operation of the Robin Rigg OWF in the northern Irish Sea, adding to the existing evidence base from other offshore wind farms in European waters and lending further support to empirical data which indicate regional variation in species‐specific avoidance responses.

## AUTHOR CONTRIBUTIONS

Gillian Vallejo, Sarah Canning, and Fiona Caryl carried out the data analysis, Emily Nelson wrote the paper with input from Kate Grellier, Ross McGregor and all other authors, and Nancy McLean had overall responsibility for the project.

## CONFLICT OF INTEREST

None declared.

## DATA ACCESSIBILITY

Robin Rigg offshore wind farm monitoring data are available free of charge via the Crown Estate Marine Data Exchange (http://www.marinedataexchange.co.uk/).
