# Supplementary material for: Responses of two marine top predators to an offshore wind farm
Source: Ecol Evol. 2017 Sep 18;7(21):8698–708. doi: 10.1002/ece3.3389 (PMC5677494; doi:10.1002/ece3.3389)

**Responses of two marine top predators to an offshore wind farm**

**Gillian C. Vallejo, Kate Grellier, Emily J. Nelson, Ross M. McGregor, Sarah J. Canning, Fiona M. Caryl & Nancy McLean.**

Supplementary online material

**Appendix S1** **Survey schedules**

Surveys included in the guillemot analysis are presented in Table S1.1 and surveys included in the harbour porpoise analysis are presented in Table S1.2. In most cases, seabirds including guillemot, and marine mammals including harbour porpoise, were recorded simultaneously. However, in some cases, only seabirds or only marine mammals were recorded resulting in differences in the survey regimes between the two species. The data analysed represent a subset of the total data collected during the monitoring program as it was necessary to exclude surveys with poor spatial coverage and surveys carried out under conditions that were not represented across all three phases. For a complete account of the data collected throughout the monitoring program, please see reports produced for Marine Scotland by the Robin Rigg Management Group (Walls *et al*. 2013a; 2013b; 2013c).

**References**

Walls, R., Canning, S., Lye, G., Givens, L., Garrett, C. & Lancaster, J. (2013a) *Analysis of Marine Environmental Monitoring Plan Data from the Robin Rigg Offshore Wind Farm, Scotland (Operational Year 1).* Natural Power report to E.ON Climate and Renewables.

Walls, R., Pendlebury, C., Lancaster, J., Lye, G., Canning, S., Malcom, F., Rutherford, V., Givens., L. & Walker, A. (2013) *Analysis of Marine Environmental Monitoring Plan Data from the Robin Rigg Offshore Wind Farm, Scotland (Operational Year 2)*. Natural Power report to E.ON Climate and Renewables.

Walls, R., Pendlebury, C., Lancaster, J., Canning, S., Lye, G., Malcom, F., Kerr, D., Givens, L. & Rutherford, V. (2013c) *Analysis of Marine Environmental Monitoring Plan Data from the Robin Rigg Offshore Wind Farm, Scotland (Operational Year 3).* Natural Power report to E.ON Climate and Renewables.

**Table S1.1** The number and timing of boat-based surveys for guillemot undertaken during the preconstruction (light grey), construction (dark grey) and operational (hatched) phases of the Robin Rigg offshore wind farm included in the final dataset.

| Year | Jan | Feb | Mar | Apr | May | Jun | Jul | Aug | Sep | Oct | Nov | Dec |
| --- | --- | --- | --- | --- | --- | --- | --- | --- | --- | --- | --- | --- |
| 2001 |  |  |  |  |  |  |  |  |  | 1 | 2 | 1 |
| 2002 | 1 | 1 | 1 | 1 | 1 | 1 |  | 1 |  | 1 | 1 | 1 |
| 2003 |  |  |  | 1 | 1 |  |  |  |  |  |  |  |
| 2004 | 1 | 1 | 1 |  | 1 |  |  | 2 | 1 |  |  |  |
| 2005 |  |  |  |  |  |  |  |  |  |  |  |  |
| 2006 |  |  |  |  |  |  |  |  |  |  |  |  |
| 2007 |  |  |  |  |  |  | 2 |  |  |  |  |  |
| 2008 | 1 | 1 | 1 | 1 | 1 | 1 | 1 | 1 | 1 | 1 | 1 | 1 |
| 2009 | 1 | 1 | 1 | 1 | 1 | 1 | 1 | 1 | 1 | 1 |  | 1 |
| 2010 | 1 | 1 | 1 | 1 | 1 | 1 | 1 | 1 | 1 |  | 1 | 1 |
| 2011 | 1 | 1 | 1 | 1 | 1 | 1 | 1 | 1 | 1 | 1 | 1 | 1 |
| 2012 | 1 | 1 |  |  |  |  |  |  |  |  |  |  |

**Table S1.2** The number and timing of boat-based surveys for harbour porpoise undertaken during the preconstruction (light grey), construction (dark grey) and operational (hatched) phases of the Robin Rigg offshore wind farm included in the final dataset.

| Year | Jan | Feb | Mar | Apr | May | Jun | Jul | Aug | Sep | Oct | Nov | Dec |
| --- | --- | --- | --- | --- | --- | --- | --- | --- | --- | --- | --- | --- |
| 2001 |  |  |  |  |  |  |  |  |  |  |  |  |
| 2002 |  |  |  |  |  |  |  |  |  |  |  |  |
| 2003 |  |  |  |  |  |  |  |  |  |  |  |  |
| 2004 |  | 1 | 1 |  |  |  |  | 2 | 1 | 1 | 1 | 1 |
| 2005 | 1 |  |  |  |  |  |  |  |  |  |  |  |
| 2006 |  |  |  |  |  |  |  |  |  |  |  |  |
| 2007 |  |  |  |  |  |  | 2 |  |  |  |  |  |
| 2008 |  | 2 | 1 | 1 | 1 | 1 | 1 | 1 | 1 | 1 | 1 | 1 |
| 2009 | 1 | 1 | 1 | 1 | 1 | 1 | 1 | 1 | 1 | 1 |  | 1 |
| 2010 |  | 1 | 1 | 1 | 1 | 1 | 1 | 1 | 1 |  | 1 | 1 |
| 2011 | 1 | 1 | 1 | 1 | 1 | 1 | 1 | 1 | 1 | 1 | 1 | 1 |
| 2012 | 1 | 1 |  |  |  |  |  |  |  |  |  |  |

**Appendix S2 Survey effort per vessel**

**Table S2.1** Guillemot survey effort (number of 600 m segments) per vessel.

| Vessel | Effort (No. of Segments) | Viewing Platform Height (m) |
| --- | --- | --- |
| *Maid Good* | 4,436 | 4.5 |
| *Pilgrim* | 485 | 4 |
| *PV Tiger* | 1,920 | 4.5 |
| *Solway Protector* | 10,591 | 4.5 |
| *Talisman of Wight* | 672 | 3.5 |

**Table S2.2** Harbour porpoise survey effort (number of 1000 m segments) per vessel.

| Vessel | Effort (No. of Segments) | Viewing Platform Height (m) |
| --- | --- | --- |
| *Maid Good* | 2,702 | 4.5 |
| *Pilgrim* | 431 | 4 |
| *PV Tiger* | 1,346 | 4.5 |
| *Solway Protector* | 4,134 | 4.5 |
| *Talisman of Wight* | 398 | 3.5 |

**Appendix S3 Harbour porpoise survey effort per sea state**

| Sea state | Effort (No. of Segments) |
| --- | --- |
| 0 | 442 |
| 1 | 915 |
| 2 | 2,813 |
| 3 | 1,827 |
| 4 | 2,500 |
| 5 | 514 |

**Appendix S4 Model formulation and parameterisation**

*Guillemot*

The negative binomial generalised additive mixed effects model (NB GAMM) used to analyse guillemot abundance and distribution data can be expressed as:

$\text{N}_{\text{ijk}\text{ }}\text{\textasciitilde}\text{ }\text{NB(}\text{μ}_{\text{ijk}}\text{, }\text{θ}\text{)}$

where the observed number of guillemot (*N*) in segment *k*, in transect *j*, in survey *i*, follow a negative binomial distribution (NB with a dispersion parameter *θ*). The full model can be written as:

$$\text{E}(\text{N}_{\text{ijk}})=\mu_{ijk}$$

$\text{var}\text{(}\text{N}_{\text{ijk}}\text{) }\text{=}\text{ }\text{μ}_{\text{ijk}}\text{ + }\frac{{\text{μ}_{\text{ijk}}}^{\text{2}}}{\text{θ}}$ $\log\left( \text{μ}_{\text{ijk}} \right)\text{= α}\text{ }\text{+}\text{ }\text{Phase}_{\text{ijk}}\text{ +}{\text{ }\text{ƒ}}_{\text{s}}\left( \text{Latitude}_{\text{ijk}} \text{,}\text{ }\text{Longitude}_{\text{ijk}} \right)\text{+ }\text{Phase}_{\text{ijk}}\text{ + }\text{a}_{\text{i}}\text{ +}{\text{ }\text{b}}_{\text{ij}}$

$\text{a}_{\text{i}}\text{ }\text{\textasciitilde}\text{ }\text{N}\text{(0, }{\text{σ}^{\text{2}}}_{\text{Survey}}\text{)}$

$\text{b}_{\text{i}\text{ }\text{j}\text{ }}\text{\textasciitilde}\text{ }\text{N}\text{(0, }{\text{σ}^{\text{2}}}_{\text{Transect}}\text{)}$

The term $\text{ƒ}_{\text{s}}\left( \text{Latitude}_{\text{ijk}\text{ }}\text{,}{\text{ }\text{Longitude}}_{\text{ijk}} \right)$ is a two-dimensional spatial smooth for phase *s*, estimated using O’Sullivan penalised splines (Wand & Ormerod 2008). $\text{a}_{\text{i}}$ is a random intercept for survey and $\text{b}_{\text{ij}}$ is a random intercept for transect within survey, included in order to model short-term spatial and temporal correlation within a survey and within a transect. Both error terms are assumed to be normally distributed.

*Harbour porpoise*

The zero-inflated Poisson generalised additive mixed effects model (ZIP GAMM) used to analyse harbour porpoise abundance and distribution data can be expressed as:

$\text{N}_{\text{ijk}\text{ }}\text{\textasciitilde}\text{ }\text{ZIP(}\text{μ}_{\text{ijk}}\text{, }\text{π}_{\text{ijk}}\text{)}$

where the observed number of harbour porpoise (*N*) in segment *k*, in transect *j*, in survey *i*, follow a zero-inflated Poisson distribution (ZIP). The Poisson part of the GAMM is represented by $\text{μ}_{\text{ijk}}$ and the binary part of the GAMM is represented by $\text{π}_{\text{ijk}}$ . The full model can be written as:

$$\text{E}(\text{N}_{\text{ijk}})={\text{(1 }-\text{ }\text{π}_{\text{ijk}})\text{ ×}\text{ }\mu}_{ijk}$$

$$\text{var}\text{(}\text{N}_{\text{ijk}}\text{) = (1 }-\text{ }\text{π}_{\text{ijk}})\text{ × }(\text{μ}_{\text{ijk}}\text{ }\text{+}\text{ }\text{π}_{\text{ijk}}\text{ × }{\text{μ}_{\text{ijk}}}^{\text{2}})$$

$\log\left( \text{μ}_{\text{ijk}} \right)\text{= α}\text{ }\text{+}\text{ }\text{Phase}_{\text{ijk}}\text{ }\text{×}{\text{ }\text{ƒ}}_{\text{s}}\left( \text{Latitude}_{\text{ijk}} \text{,}\text{ }\text{Longitude}_{\text{ijk}} \right)\text{+ }\text{Phase}_{\text{ijk}}\text{ + }\text{a}_{\text{i}}\text{ +}{\text{ }\text{b}}_{\text{ij}}$

$$\text{logit }\left( \text{π}_{\text{ijk}} \right)\text{ }\text{=}\text{ }\text{γ}\text{ +}{\text{ }\text{Sea}\text{ }\text{State}}_{\text{ijk}}$$

$\text{a}_{\text{i}}\text{ }\text{\textasciitilde}\text{ }\text{N}\text{(0, }{\text{σ}^{\text{2}}}_{\text{Survey}}\text{)}$

$\text{b}_{\text{ij}\text{ }}\text{\textasciitilde}\text{ }\text{N}\text{(0, }{\text{σ}^{\text{2}}}_{\text{Transect}}\text{)}$

The term $\text{ƒ}_{\text{s}}\left( \text{Latitude}_{\text{ijk }}\text{,}\text{ Longitude}_{\text{ijk}} \right)$ is a two-dimensional spatial smooth for phase *s*, estimated using O’Sullivan penalised splines (Wand & Ormerod 2008). $\text{a}_{\text{i}}$ is a random intercept for survey and $\text{b}_{\text{i j}}$ is a random intercept for transect within survey, included in order to model short-term spatial and temporal correlation within a survey and within a transect. Both error terms are assumed to be normally distributed.

Both models were implemented within a Bayesian framework. Within this framework, posterior distributions for each parameter were generated using Markov Chain Monte Carlo simulation. In instances where previous knowledge is available regarding model terms, prior distributions can be specified based on this knowledge; however in this case, no prior expectations were applied to the model, and so-called ‘uninformative’ priors were specified.

Markov Chain Monte Carlo (MCMC) is a computational technique which can be used for Bayesian inference. This algorithm simulates chains of values for which any given value is related only to the value immediately preceding it. These values form the posterior probability distributions of the model terms. After an initial ‘burn in’ period during which the values move towards the optimal region for the probability distribution, the values should be accurately sampling the probability distribution of the model terms. By convention in Bayesian inference, three independent MCMC chains are generated simultaneously, and the validity of the computations can therefore be assessed by whether or not the chains have converged to similar optima. This is assessed visually and is known as ‘mixing’.

Analyses were carried out using JAGS (Plummer 2003) implemented through the R package ‘R2jags’ (Yu-Sung & Masanao 2015) in R version 3.2.2 (R Core Development Team 2015). To facilitate computations, continuous covariate data (longitude and latitude) were rescaled prior to analysis so that values fell within a range of -1 to +1 with a mean of zero (but without distorting the spatial distance between the sampling locations). Diffuse (uninformative) priors were selected for all parameters. Gaussian distributed priors with a mean of zero and a precision of 0.0001 were used for the Poisson intercepts, the binary intercepts and for parameters associated with phaseand sea state. All other priors followed Gaussian distributions with a mean of zero and a standard deviation following a half-Cauchy (25) or half-Cauchy (5) distribution (for the harbour porpoise and guillemot models respectively). These priors were selected since they have been found to improve mixing (Gelman 2006; Marley & Wand 2010). For each model, posterior distributions of parameters were calculated from a total of 30,000 saved iterations originating from three chains and incorporating a thinning rate of 10 (i.e. only every tenth iteration was saved, eliminating any dependency structure in the samples). Each chain was allowed a burn-in of 71,000 iterations. Mixing was good for all parameters for the harbour porpoise model and for all but the coefficients for phase in the guillemot model, for which mixing was reasonable. Investigation of posterior mean Pearson’s residuals provided no evidence of over-dispersion or residual spatio-temporal autocorrelation for either model. In order to assess whether the models fit the data, we simulated data from the model and compared this to the observed data. To be more precise, posterior predicted values were calculated from models generated by each Markov Chain Monte Carlo (MCMC) iteration and these were used to assess the fit of the model to the raw data. The Bayesian *p*-value, calculated by comparing the proportion of iterations for which the sums of squares of the Pearson residuals of the posterior predicted values are greater than the sums of squares of the residuals of the observed data, which would be expected to occur ~50% of the time for a perfectly fitting model, suggested that models fitted the data appropriately.

**References**

Gelman, A. (2006) Prior distributions for variance parameters in hierarchical models (comment on article by Browne and Draper). *Bayesian analysis*, 1, 515-534.

Marley, J. & Wand, M. (2010) Non-standard semiparametric regression via BRugs. *Journal of Statistical Software*, 37, 1 – 30.

Plummer, M. (2003) [*JAGS: A Program for Analysis of Bayesian Graphical Models Using Gibbs Sampling*](http://www.ci.tuwien.ac.at/Conferences/DSC-2003/Proceedings/Plummer.pdf)*. Proceedings of the 3rd International Workshop on Distributed Statistical Computing (DSC 2003), March 20–22, Vienna, Austria*. ISSN 1609-395X.

R Core Development Team (2015) *R: A language and environment for statistical computing*. R Foundation for Statistical Computing, Vienna, Austria.

Wand, M. P. & Ormerod, J. T. (2008) On semiparametric regression with O'Sullivan penalized splines. *Australian & New Zealand Journal of Statistics*, 50, 179-198.

Yu-Sung, S. & Masanao, Y. (2015) *R2jags: Using R to Run 'JAGS'.* R package version 0.5-7

**Appendix S5 Mean observations per segment per month for (a) guillemots and (b) harbour porpoise with standard errors.**

(a)


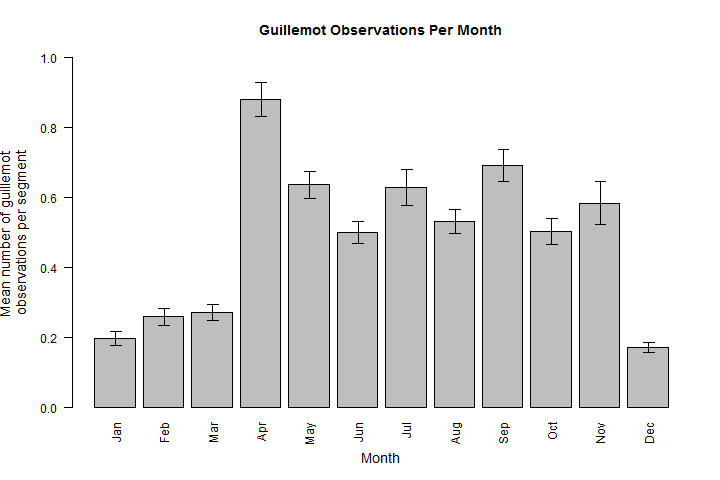


(b)


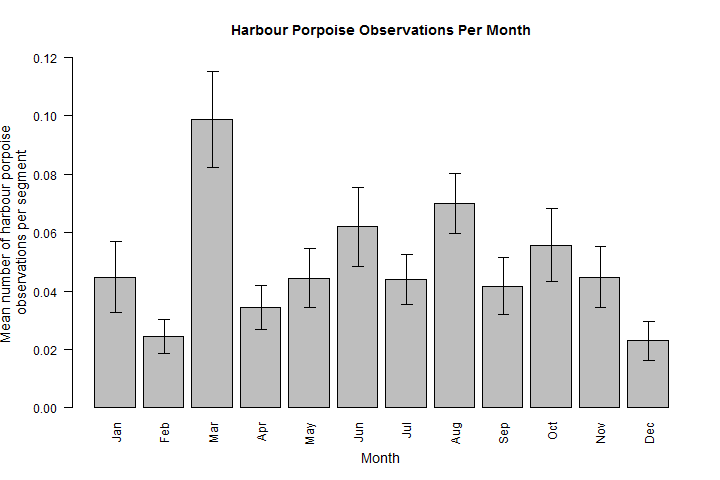

Supplement: Supplementary file 1 [file ECE3-7-8698-s001.docx]
